# Nuts4Brain-RCT: Protocol for a Three-Arm Randomized Controlled Trial Examining the Dose–Response Effects of Nut Consumption on Mental Health in Young Adults

**DOI:** 10.3390/healthcare14040435

**Published:** 2026-02-09

**Authors:** Arthur Eumann Mesas, Fernando Peral-Martínez, Tomás Olivo-Martins-de-Passos, Estela Jiménez-López, Nuria Beneit, Isabel Antonia Martínez-Ortega, Valentina Díaz-Goñi, Sandra Herraiz-Garrote, Irene Martínez-García, David Casanova-Rodríguez, Eva Rodríguez-Gutiérrez, Bruno Bizzozero-Peroni, Ana Ballesta-Castillejos, Shkelzen Cekrezi, Irene Marcilla-Toribio, Sergio Núñez de Arenas-Arroyo, Carlos Álvarez-Herraiz, Carla Soraya Costa Maia, Soraia Pinheiro Machado, Luis Felipe Nunes de Oliveira, Sandra Serrano-Martínez, Vanessa Martínez-Madrid, María Concepción Calderón-Alva, José Francisco López-Gil, Rubén Fernández-Rodríguez, Cristina Cadenas-Sánchez, María Medrano, María Eugenia Visier-Alfonso, Vicente Martínez-Vizcaíno, Miriam Garrido-Miguel

**Affiliations:** 1 Health and Social Research Center, Universidad de Castilla-La Mancha, 13071 Cuenca, Spain; arthur.emesas@uclm.es (A.E.M.); fernando.peral@uclm.es (F.P.-M.); tomas.olivo@uclm.es (T.O.-M.-d.-P.); profesor.nbeneit@uclm.es (N.B.); isabela.martinez@uclm.es (I.A.M.-O.); valentina.diaz@uclm.es (V.D.-G.); sandra.herraiz@uclm.es (S.H.-G.); davidcasanovafisio@gmail.com (D.C.-R.); eva.rodriguez@uclm.es (E.R.-G.); bruno.bizzozero@uclm.es (B.B.-P.); ana.ballesta@uclm.es (A.B.-C.); shkelzen.cekrezi@uclm.es (S.C.); irene.marcilla@uclm.es (I.M.-T.); sergio.nunezdearenas@uclm.es (S.N.d.A.-A.); mariaeugenia.visier@uclm.es (M.E.V.-A.); vicente.martinez@uclm.es (V.M.-V.); miriam.garrido@uclm.es (M.G.-M.); 2 Centro de Investigación Biomédica en Red de Salud Mental (CIBERSAM), Instituto de Salud Carlos III, 28029 Madrid, Spain; 3 Facultad de Enfermería de Albacete, Universidad de Castilla-La Mancha, 02071 Albacete, Spain; irene.mgarcia@uclm.es; 4 Department of Nursing, Pharmacology and Physiotherapy, Faculty of Medicine and Nursing, University of Córdoba, 14071 Cordoba, Spain; 5 Instituto de Investigación Sanitaria de Castilla la Mancha (IDISCAM), 45071 Toledo, Spain; 6 Higher Institute of Physical Education, Universidad de la República, Rivera 40000, Uruguay; 7 Aging Research Center, Department of Neurobiology, Care Sciences and Society, Karolinska Institutet and Stockholm University, SE-17177 Stockholm, Sweden; 8 Consejería de Educación, Junta de Comunidades de Castilla-La Mancha, 16071 Cuenca, Spain; ccah21@educastillalamancha.es; 9 Health Science Center, Universidade Estadual do Ceará, Fortaleza 60714-903, CE, Brazil; carla.maia@uece.br (C.S.C.M.); soraia.machado@uece.br (S.P.M.); felipee.oliveira@aluno.uece.br (L.F.N.d.O.); 10 Servicio de Salud de Castilla-La Mancha (SESCAM), 16071 Cuenca, Spain; sandras@sescam.jccm.es (S.S.-M.); vamarma@hotmail.com (V.M.-M.); ccalderona@sescam.jccm.es (M.C.C.-A.); 11 School of Medicine, Universidad Espíritu Santo, Samborondón 092301, Ecuador; josefranciscolopezgil@gmail.com; 12 Vicerrectoría de Investigación y Posgrado, Universidad de Los Lagos, Osorno 5290000, Chile; 13 Department of Physical Education and Sports, Faculty of Sports Science, Sport and Health University Research Institute (iMUDS), University of Granada, 18007 Granada, Spain; r.fernandezrodriguez@deakin.edu.au (R.F.-R.); cadenas@go.ugr.es (C.C.-S.); 14 Food & Mood Centre, Institute for Mental and Physical Health and Clinical Translation (IMPACT), Deakin University, Geelong 3220, VIC, Australia; 15 The Grounded Minds Consortium, A Deakin University & Flinders University Collaboration, Geelong 3220, VIC, Australia; 16 Centro de Investigación Biomédica en Red Fisiopatología de la Obesidad y Nutrición (CIBERobn), Instituto de Salud Carlos III, 18007 Madrid, Spain; maria.medrano@unavarra.es; 17 Institute for Innovation & Sustainable Development in Food Chain (IS-FOOD), Campus de Arrosadía, Public University of Navarra, 31009 Pamplona, Spain; 18 Faculty of Health Sciences, Universidad Autónoma de Chile, Talca 3465548, Chile

**Keywords:** nuts, mental health, depression, cognitive function, young adults, RCT, study protocol

## Abstract

**Introduction**: Mental disorders, particularly depression and anxiety, represent a leading source of morbidity and disability in young adults, a group increasingly exposed to cumulative psychological processes and environmental stressors. Although evidence links healthy dietary patterns with improved mental health symptoms, the specific contribution of nut consumption (rich in anti-inflammatory, antioxidant, and neuroprotective compounds) remains insufficiently examined in this population. Current evidence also lacks robust data on the dose–response effects of nut consumption and mechanistic pathways involving biomarkers relevant to brain function and mental health. The Nuts4Brain-RCT will investigate the impact of a 6-month mixed-nut consumption intervention on mental health symptoms, cognitive functioning, sleep quality, overall well-being, health-related quality of life, and biochemical brain function mediators in young adults. **Methods and analysis**: A 6-month, randomized, single-blinded, three-arm, dose–response, parallel-group clinical trial will be conducted with 135 university students aged 18–24 years from the University of Castilla-La Mancha, Cuenca, Spain. The participants will be randomly assigned in a 1:1:1 ratio to one of three groups: (1) a control group maintaining their habitual diet while avoiding nut consumption; (2) a 30 g/day mixed-nut consumption group; or (3) a 60 g/day mixed-nut consumption group. The nut consumption intervention will include unsalted, unroasted walnuts, almonds, hazelnuts, and pistachios. The primary outcomes will include depressive (BDI-II) and anxiety (GAD-7) symptoms, perceived stress (PSS-10), cognitive performance (NIH Toolbox), and plasma brain-derived neurotrophic factor (BDNF). Assessments will take place at baseline and at 3 (intermediate), 6 (end of intervention) and 12 months (follow-up). Repeated-measures mixed-effects models will be applied to estimate the effects of nut consumption and dosage on mental health outcomes. **Ethics and dissemination**: The study adheres to the principles of the Declaration of Helsinki and the Singapore Statement on Research Integrity and obtained approval from the Clinical Research Ethics Committee of the Virgen de la Luz Hospital, Cuenca, Spain (REG: 2025/E0125). The findings will be disseminated through high-impact open-access peer-reviewed publications, presentations at scientific conferences, and social media platforms. **Trial registration**: ClinicalTrials.gov. **Identifier**: NCT07292610.

## 1. Introduction

Mental disorders, especially depression and anxiety, are among the leading causes of morbidity and disability in young people worldwide [1,2]. According to estimates from the Global Burden of Disease Study, in 2023, approximately 322 million individuals were living with depressive disorders and 470 million with anxiety disorders, representing the second and sixth leading contributors, respectively, to the overall disease burden among youths aged 10–24 years [1,2]. Recent trend analyses underscore the growing urgency of addressing mental health conditions among young people, revealing a significant upward trajectory [1,3,4]. This increase has been linked to a combination of cumulative stressors, including academic pressures, social expectations, economic uncertainty, and the pervasive influence of digital technology, which have been further intensified by the coronavirus disease 2019 (COVID-19) pandemic [5]. In Spain, suicide has become the leading cause of death among individuals aged 12–29 years, and recent government surveys indicate that nearly half of university students (49.4%) experience depressive symptoms [6,7]. These findings highlight the critical need for timely, comprehensive, and targeted intervention strategies for this vulnerable population [8].

Despite the urgent need for effective management, depression and anxiety remain difficult to address because of their multifactorial etiology, which is characterized by a dynamic interplay of biological, psychosocial, and environmental factors [9]. Conventional pharmacological treatments, such as antidepressants and anxiolytics, often have limited efficacy and may be accompanied by undesirable side effects [10], highlighting the need for alternative or complementary nonpharmacological therapeutic approaches, such as promoting healthy lifestyles, including a healthy diet [10]. In this context, scientific evidence from observational [11] and clinical trials [12] has shown that healthy dietary patterns, including the Mediterranean diet, are consistently associated with reduced risks of depression and the alleviation of its symptoms [13,14]. While general dietary improvements are beneficial, understanding the specific impact of key nutrient-dense foods is vital for designing targeted and effective interventions [15]. Specifically, nuts may play a crucial role in healthy dietary patterns that have been associated with lower rates of depression [16,17,18] and improved mood in the general population. Nuts are characterized by their high nutrient density and are rich in bioactive compounds with anti-inflammatory, antioxidant, and neuroprotective potential [19,20]. They comprise a diverse range of varieties, including walnuts, almonds, hazelnuts, pistachios, and others. Nutritionally, they provide substantial amounts of monounsaturated fatty acids (MUFAs) and polyunsaturated fatty acids (PUFAs), including omega-9 oleic acid, the essential fatty acids omega-3 alpha-linolenic acid, and omega-6 linoleic acid, as well as high-quality plant protein, fiber, polyphenols, melatonin, vitamins, and minerals [19,21,22].

The proposed mechanisms indicate that nut compounds may alleviate depressive and anxiety symptoms through multiple pathways, including reducing systemic inflammation, attenuating oxidative stress, and modulating the gut-brain axis [23,24]. Moreover, the benefits of nut consumption may extend to other health-related aspects. Preliminary evidence suggests that nuts may increase sleep quality, which is likely attributable to their melatonin content [25], a neurohormone critical for regulating the sleep–wake cycle [26]. In terms of cognitive function, high levels of PUFAs and antioxidants in nuts may improve vascular elasticity and endothelial function in cerebral vessels and have immunomodulatory properties, which potentially promote neurogenesis and neuroplasticity [24,27]. Brain-derived neurotrophic factor (BDNF) has received increasing attention as the most abundant and widely expressed neurotrophin in the central nervous system and plays an important role in neural and cognitive function [28]. Peripheral BDNF levels have been associated with several mental health conditions, such as depressive disorders, anxiety, insomnia, eating disorders, and cognitive impairment [29,30,31,32]. BDNF levels are potentially modifiable through lifestyle [33] with some diet patterns, such as Mediterranean, showing increased circulating levels [34,35]. However, it has not yet been demonstrated whether the relationships between nut consumption and mental health or cognitive function are indeed mediated by biomarkers of inflammation, oxidative stress, BDNF [36], and other cardiometabolic risk factors, such as blood pressure and adiposity.

In this line, the current evidence base remains limited, methodologically heterogeneous, and insufficient for establishing robust dietary recommendations. It is still uncertain whether the observed benefits of nuts on mental health outcomes can be attributed to nut consumption per se or are instead confounded by cooccurring health-promoting behaviors, such as higher overall dietary quality, greater engagement in physical activity [37], better sleep patterns [38] and improved weight management [39], which are frequently reported among habitual nut consumers. Moreover, although a daily intake of 30 g is generally regarded as the minimum effective dose for cardiovascular protection [40], the potential existence of a dose–response relationship in relation to mental health outcomes has not been systematically investigated. This evidentiary gap highlights the need for rigorously designed randomized controlled trials (RCTs) that incorporate extended follow-up periods to generate robust empirical data capable of informing specific and evidence-based recommendations regarding the potential effects of nut consumption on mental health. In addition, the predominance of studies conducted in older adults, largely within the context of preventing cognitive decline [41], highlights a substantial gap in evidence concerning younger adult populations.

Early adulthood is characterized by substantial physical, psychological, and social transitions and frequently coincides with the onset of mental health problems [42,43]. This developmental stage merits particular attention, as early manifestations of mental health symptoms are closely associated with adverse physical and psychological outcomes later in life [44]. Furthermore, lifestyle behaviors established during this period exert a sustained influence on subsequent mental health trajectories [45,46]. Within this context, developing a more comprehensive understanding of the relationship between nut consumption and mental health symptoms in early adulthood is essential for informing evidence-based strategies aimed at promoting mental well-being across the lifespan.

The Nuts4Brain-RCT provides several distinctive contributions beyond existing trial designs. In contrast to previous studies, which have primarily focused on preventing cognitive decline and dementia in older adults [37,47] or have relied on observational evidence [17,18], this protocol targets young adults aged 18–24 years, a critical developmental period for mental health [48,49]. Regarding the three available RCTs on nut consumption and mental health associations in young populations, Pribis et al. [50] examined the effects of walnut consumption (1/2 cup or 60 g) on mood and cognitive performance among young adults. They found a significant medium effect size improvement in mood among males (Cohen’s d = 0.708, *p* = 0.043), but no significant changes were found for females or when both sexes were combined, as observed in another article with data from the same RCT [51]. Additionally, an increase in inferential verbal reasoning (d = 0.567; *p* = 0.009) was found, but no significant increases were detected in memory or non-verbal reasoning ability [50]. Herselman et al. (2022) [48] examined the effects of daily walnut consumption (56 g/day for 16 weeks) and academic stress on mental health in university students. A significant decrease in feelings associated with depression was found in women who consumed walnuts compared to controls; however, male data were not analyzed due to low participant numbers. In addition, Pinar-Martí et al. [49] studied the effect of walnut consumption (30 g/day for six months) on neuropsychological development in adolescents. Although no improvements in neuropsychological function were found when comparing the intervention and control groups, some benefits were observed in variables such as sustained attention and fluid intelligence among participants with better adherence to the intervention. The key methodological innovation is the dose–response design (30 g/day vs. 60 g/day), addressing the current lack of evidence on optimal nut intake for mental well-being. Moreover, whereas many prior trials have assessed isolated outcomes, this study adopts a comprehensive and integrative approach by combining psychometric assessments, objective cognitive testing, and biological markers related to underlying mechanisms. Finally, the inclusion of a 12-month follow-up (comprising a 6-month intervention and a 6-month post-intervention period) allows for the evaluation of the sustainability of effects, thereby distinguishing this protocol from shorter-term interventions.

Therefore, the Nuts4Brain-RCT study aims to evaluate the impact of a 6-month intervention involving nut consumption on depressive and anxiety symptoms, perceived stress, cognitive functioning, sleep quality, overall well-being, health-related quality of life, and biochemical mediators of brain function in young adults. The present manuscript constitutes a pre-results protocol publication documenting the study design, procedures, and analytical plan prior to participant recruitment and data collection.

## 2. Methods

This randomized controlled trial protocol is derived from the “Nuts4Brain project: The relationship between nut consumption and mental health outcomes during adulthood” (reference PI21/01898), co-funded by the European Union and the Carlos III Health Institute, Spain.

The study was designed in accordance with the Standard Protocol Items: Recommendations for Interventional Trials (SPIRIT) guidelines [52,53], which offer a rigorous framework for the development and transparent reporting of clinical trial protocols.

### 2.1. Ethical Aspects

The Nuts4Brain-RCT study protocol adhered to the principles of the Declaration of Helsinki [54] and the Singapore Statement on Research Integrity [55] and obtained approval from the Clinical Research Ethics Committee of the Virgen de la Luz Hospital (REG: 2025/E0125) and has been registered at ClinicalTrials.gov (NCT07292610). All interested participants will be required to sign an informed consent form prior to participation in the study.

### 2.2. Study Design

A 6-month, randomized, single-blinded, three-arm parallel-group clinical trial will be conducted in young adults (aged 18–24 years). The participants will be randomly assigned in a 1:1:1 ratio to one of three groups: (1) a control group maintaining their habitual diet while avoiding nut consumption; (2) a 30 g/day mixed-nut consumption group; or (3) a 60 g/day mixed-nut consumption group while avoiding nut consumption from other sources.

**Figure 1:**
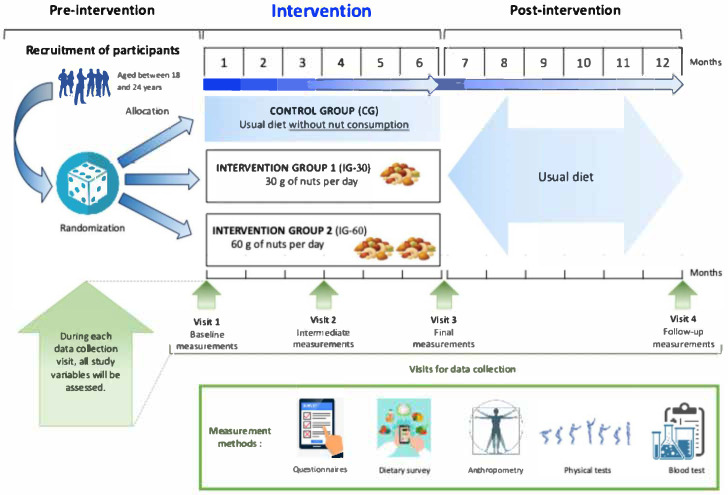
Planned study design.

As illustrated in Figure 1, outcomes and other relevant variables will be assessed at baseline, 3 months after the beginning of the intervention, 6 months (i.e., at the end of the intervention), and 6 months after the end of the intervention, resulting in a total of data collection points over 12 months.

### 2.3. Eligibility Criteria

The inclusion criteria will be as follows: (1) being enrolled as a university student in any undergraduate academic program at the University of Castilla-La Mancha (UCLM), campus of Cuenca; (2) aged between 18 and 24 years; and (3) being willing and able to provide written informed consent to participate.

The exclusion criteria will include any known food allergies, particularly tree nuts or peanuts; chronic psychiatric or gastrointestinal medical conditions diagnosed and/or under pharmacological treatment; and the expected inability to attend follow-up visits because of planned internship abroad or study completion during the trial period. Those who are pregnant at the time of randomization or who plan to become pregnant during the follow-up period will be ineligible.

### 2.4. Sample Size Calculation

The sample size was calculated using the Grayling and Wason method for multi-arm trials [56], which is appropriate for a design with two experimental groups and one control group. A medium effect size (Cohen’s d = 0.5) and homogeneity of variance (σ = 1) were assumed across groups as recommended [57] when no clear estimate on this association is provided in the available studies with similar design [48,49]. With a two-sided α of 0.05 and 80% power (β = 0.20), the required sample size was obtained via the “des_ss_norm()” function in R statistical software, version 4.5.2 (R Core Team, 2024, Vienna, Austria). All three arms were allocated at a 1:1:1 ratio. To account for multiple comparisons, the Benjamini–Hochberg correction was applied to control the false discovery rate. The power calculation was based on a disjunctive criterion, which estimates the probability of detecting at least one significant difference between any intervention group and the control group. The initial calculation yielded a requirement of 42 participants per group. After accounting for an anticipated 20% dropout rate, the final sample size was adjusted to 53 participants per group. This resulted in a total target enrollment of 159 participants across all three study arms. The participants will initially be drawn from the 463 students enrolled in the Nuts4Brain-Z observational study (data collection September–December 2023) [58,59,60]. If necessary, recruitment will be extended to additional UCLM undergraduate students to reach the target sample size.

The sample size calculation was based exclusively on the primary outcome as we did not find previous studies examining the association between nut consumption and our secondary outcomes that would provide the necessary effect size estimates for power and sample size calculations. Consequently, the study should be considered exploratory with regard to these secondary outcomes, and any findings related to them should be interpreted with appropriate caution. While our sample size provides adequate power for detecting changes in the primary outcome, we acknowledge that it may be underpowered for detecting meaningful differences in secondary outcomes, particularly if the true effects are small to moderate in magnitude.

### 2.5. Participant Recruitment

Following ethics approval, authorization will be obtained from university authorities at UCLM in Cuenca, Spain. Professors will be contacted to request 15 min of class time for a brief presentation about the study, during which flyers will be distributed. Interested students can provide contact information either by giving their mobile number or scanning a QR code to complete an online form. QR codes will also be available on posters throughout the university. A research team member will then contact interested students via WhatsApp LLC (Meta Way, CA, USA) to answer questions and, if they agree to participate, schedule the baseline visit. All participants will be instructed to avoid nut consumption for at least 7 days before the baseline assessment.

### 2.6. Randomization and Allocation Processes

The randomization and allocation processes will occur before the baseline assessment. To ensure balance across study arms, randomization will be stratified by sex considering the ratio of 2 males:3 females, since this is the ratio observed in the reference population of university students on the campus of Cuenca of the UCLM. Allocation concealment will be maintained using sequentially numbered, opaque, sealed envelopes, which will be opened only at the point of assignment. To minimize selection bias, the allocation sequence will remain concealed from the research team until the end of the intervention (six months). Participants will be informed of their group allocation immediately after the randomization procedure has been completed.

### 2.7. Intervention Implementation

Once randomization is complete, participants will receive printed and verbal standardized instructions to maintain their usual lifestyle habits throughout the study period, except for nut consumption, where the following group-specific instructions will be provided:

- Control group (CG): Participants will be instructed to continue their habitual diet while avoiding direct nut consumption, although the sporadic consumption of foods that may contain trace amounts of nuts as ingredients (e.g., baked goods, ice cream) does not constitute a protocol violation.
- Intervention groups (IG-30 and IG-60): Participants will be instructed to consume 30 g or 60 g of unsalted, unroasted mixed nuts daily and to exclusively consume the amount of nuts provided by the researchers, refraining from consuming other sources of nuts. For the participants in the CG, sporadic exposure to trace amounts of nuts in processed foods (e.g., baked goods, ice cream) will be acceptable.

The nuts (a mixture of almonds, walnuts, pistachios, and hazelnuts) will be provided free of charge, in daily portions, to participants in the intervention groups for the full duration of the trial. The participants will be asked to incorporate nuts into their usual diet and to consume them in any uncooked manner they prefer, such as alone (only nuts), with salad, or yogurt.

At months 2 and 4, participants will be instructed to collect the prepacked bags containing the daily portions of nuts required for each intervention group during the subsequent period (i.e., participants in IG-30 will receive 30 g bags, and those in IG-60 will receive 60 g bags). To maintain a single-blind design, the nuts will be provided in person for each participant by a researcher who is not involved in clinical assessments. The bags containing the daily content of nuts will be specifically produced for the trial in accordance with established quality standards and sealed under a protective atmosphere. Each bag will contain a fixed mixture of nuts in the following proportions: 35% almond, 35% walnut, 20% unshelled pistachios, and 10% hazelnut. These nut mix proportions are based on the most consumed nut types in Spain and were designed to provide a balanced profile of fatty acids, micronutrients, and bioactive compounds while reflecting habitual intake, improving palatability and adherence, and facilitating translation into practical dietary recommendations.

### 2.8. Trial Monitoring

Every two weeks, all participants will receive a telephone message via WhatsApp from a member of the research team to reinforce adherence and monitor any adverse effects potentially associated with the intervention. If a participant experiences a suspected allergic reaction, intolerance, or digestive discomfort, the intervention will be immediately and permanently discontinued, and medical evaluation will be recommended.

Intervention compliance will be assessed according to the participants’ report of the consumption of at least 80% of the nut doses provided [61]. In addition, to provide an objective measure of adherence to the nut consumption protocol, blood concentrations of vitamin E (α-tocopherol) will be assessed in the four evaluations. Although the expected magnitude of increase in vitamin E associated with nut consumption varies by nut type, dose, and baseline levels, the available evidence has shown a 5 to 15% increase in plasma α-tocopherol with regular consumption of 30–80 g nuts/day over several weeks to month [62].

### 2.9. Data Collection and Clinical Assessments

Data collection and clinical assessments will be centralized at the MOVIFitness laboratory (UCLM, Cuenca) to ensure the standardization of all operational procedures. The protocol allows for the scheduling of a maximum of 15 participants per weekday, with sessions lasting approximately 3 h. These visits include the collection of venous blood samples following at least 12 h of overnight fasting, physical examinations, and cognitive testing guided by a trained multidisciplinary research team. To ensure data integrity and minimize transcription errors, validated Spanish versions of the instruments will be administered via a touchscreen tablet interface using the secured Qualtrics platform (Qualtrics, Provo, UT, USA), which facilitates both participant confidentiality and logistical portability within the clinical environment.

A food frequency questionnaire will be provided in printed format to allow for scanning. For all study variables, the participants will be informed about the procedure of the tests and instructed by a multidisciplinary research team.

### 2.10. Study Variables

Table 1 presents a summary description of the variables collected and the instruments used.

The established cutoff points for specific variables within this protocol are not rigidly fixed and possess inherent flexibility. These thresholds are subject to potential future modifications in subsequent research endeavors. Such adjustments will be driven primarily by the specific aims and hypotheses proposed by the research team, ensuring optimal alignment with evolving research objectives.

The same data collection protocol (including all study variables) applied at baseline (visit 1) will be applied at the 3-month follow-up (intermediate visit 2) and the 6-month post-intervention assessment (final intervention visit 3). Upon completion of the intervention, participants will be instructed to follow their dietary and lifestyle habits without specific restrictions. They will be invited to attend a final data collection session at 12 months (visit 4), i.e., 6 months after visit 3. Once the results of each assessment are available, participants will receive recommendations to consult their primary care physician or emergency services, if necessary.

#### 2.10.1. Primary Mental Health Outcome Measures

Depressive symptoms: The assessment of depressive symptomatology will be conducted using the Beck Depression Inventory–II (BDI-II) [63], a widely validated 21-item self-report measure designed to evaluate the severity of depressive symptoms over the preceding two weeks. Each item offers four response options scored from 0 to 3, with higher scores reflecting greater depressive severity. The BDI-II is considered the gold-standard screening instrument for individuals aged 13 years and older [64]. The Spanish version of the BDI-II has previously been validated [64] and has demonstrated good internal consistency (Cronbach’s α = 0.89) and strong construct and criterion validity across university students [65]. The total scores range from 0–63 and are derived from the sum of all 21 items. Conventional cutoffs classify symptom severity as minimal or absent (0–13), mild (14–19), moderate (20–28), or severe (29–63).

Anxiety symptoms: Anxiety symptoms will be assessed using the Generalized Anxiety Disorder 7-item scale (GAD-7) [66], a validated self-report instrument developed to screen for and quantify the severity of generalized anxiety symptoms over the preceding two weeks. The participants rated each item on a four-point scale ranging from 0 (“not at all”) to 3 (“nearly every day”), yielding a total score between 0 and 21. Higher scores indicate greater anxiety symptomatology, with established cutoffs categorizing symptom severity as mild (5–9), moderate (10–14), or severe (15–21) proposed [66]. The Spanish version of the GAD-7 [67] shows excellent reliability values (Cronbach’s α = 0.94) and good accuracy in identifying cases of GAD (sensitivity and specificity > 0.80) [66,67], including among university students (Cronbach’s α = 0.90) [68].

Stress: Perceived stress levels will be measured using the 10-item Perceived Stress Scale (PSS-10) [69,70], a widely validated self-report instrument designed to assess the degree to which individuals appraise situations in their lives as stressful over the previous month. Each item is rated on a 5-point Likert scale ranging from 0 (“never”) to 4 (“very often”), resulting in a total score between 0 and 40, where higher scores indicate greater perceived stress. Validation studies have reported satisfactory internal consistency for the Spanish version of the PSS-10 (α = 0.82) [70]. Since no validated cutoff scores exist for the PSS-10, it will be analyzed as a continuous variable, and the study sample will be classified into tertiles for comparative analysis.

Cognition: Cognitive performance will be evaluated at the 4 assessments with the National Institutes of Health (NIH) Toolbox Cognition Battery (Chicago, IL, USA), which is administered on an iPad by trained personnel. This widely used computer-based method has been normed for children and adults aged 3–85 years [71]. Specifically, the Fluid Cognition Composite Score will be measured [71], as it is more sensitive to changes resulting from lifestyle interventions than is crystallized cognition [72]. This composite score assesses executive function, processing speed, working memory, episodic memory, and attention through five individual test instruments (~30 min), including the Flanker Inhibitory Control and Attention Test (Flanker) (inhibitory control and attention), the Dimensional Change Card Sort (DCCS) (cognitive flexibility), the Picture Sequence Memory Test (PSMT) (episodic memory), the List Sorting Working Memory Test (LSWM) (working memory), and the Pattern Comparison Processing Speed Test (PCPST) (processing speed). Equivalent alternate forms of the subtests will be administered during follow-up assessments to minimize practice effects. Age-standardized scores for each evaluation and the fluid composite score will be used as the main outcomes of cognitive function. The age-adjusted fluid cognition composite score of the Spanish NIH Toolbox has shown good reliability (ω = 0.78) [73] and moderate to strong convergent validity with established neuropsychological measures [73].

#### 2.10.2. Secondary Mental Health Outcome Measures

Disordered eating behaviors: The SCOFF questionnaire [74] will be used to detect the possible presence of disordered eating. This five-item tool assesses core behaviors and attitudes related to food, weight, and body image. Responses will be recorded as “yes” or “no”, with a score of two or more indicating a probable risk of an eating disorder. The Spanish version of the SCOFF questionnaire demonstrated excellent psychometric properties among youth populations; a cutoff of more than two positive responses provided a sensitivity of 97.7% and a specificity of 94.4% for the detection of eating disorders [74].

Body image perception: Body image dissatisfaction will be assessed using the Standard Figural Stimuli (SFS), which was originally developed by Stunkard et al. [75] and subsequently validated in Spanish-speaking young populations by Rueda-Jaimes et al. [76]. The SFS consists of nine male silhouettes and nine female silhouettes arranged in ascending order of body mass index, with the numeric values concealed from the participants. Each student will first choose the figure they believe best represents their current body shape and then the figure they consider most desirable. Body image dissatisfaction is quantified as the numerical difference between the selected current and ideal silhouettes.

Resilience: The Spanish adaptation of the CD-RISC-10 [77], a shortened version of the Connor-Davidson Resilience Scale [78], will be used to assess psychological resilience. This instrument comprises ten items, with each item rated on a five-point Likert scale ranging from 0 to 4. The total scores range from 0–40, with higher scores reflecting greater levels of resilience. The scale provides a reliable and efficient measure of participants’ ability to cope with stress and adapt to adversity. This scale has shown good internal consistency (Cronbach’s α = 0.85) in young adults [77].

Health-related quality of life: Health-related quality of life (HRQoL) will be assessed using the 12-item Short Form Health Survey (SF-12), a validated instrument providing a reliable measure of perceived health status [79]. The twelve items assess functional status, well-being, and overall health across eight domains. Responses will be scored using standard algorithms [79] to generate a Physical Component Summary (PCS) and a Mental Component Summary (MCS), both of which are standardized to a mean of 50 and a standard deviation of 10. Higher scores indicate better perceived HRQoL. The SF-12 demonstrated acceptable reliability for both physical and mental component scores (reliability coefficients ≈ 0.78–0.85) [80].

Sleep quality: Sleep quality will be assessed via the Pittsburgh Sleep Quality Index (PSQI) [81], a widely validated self-report questionnaire designed to evaluate overall sleep quality over the past month. The PSQI includes 19 items that generate seven component scores: subjective sleep quality, sleep latency, sleep duration, habitual sleep efficiency, sleep disturbances, sleep medication use, and daytime dysfunction. Each component is scored from 0 to 3, with higher scores indicating poorer sleep quality. The sum of the component scores yields a global score ranging from 0 to 21, where a score greater than 5 is indicative of poor sleep quality. The Spanish version of the PSQI showed good psychometric properties (Cronbach’s alpha = 0.72) across diverse populations, including young adults and university students [82].

#### 2.10.3. Potential Confounding, Mediating, and Moderating Factors

Socioeconomic level, demographic features, and medical history: Family socioeconomic status (SES) will be derived from data concerning maternal and paternal education and occupation. Parental education will be categorized into three levels: primary (incomplete primary education or functional illiteracy), secondary (completed primary and secondary/high school education), and university (bachelor’s degree or postgraduate qualification). Paternal and maternal occupation will be classified into five hierarchical categories: (1) supervisor/manager or self-employed with ≥10 employees; (2) supervisor/manager or self-employed with <10 employees; (3) self-employed without staff; (4) unqualified staff and unskilled workers; and (5) household duties, unemployed, or other. A composite SES index will be subsequently calculated by combining parental education and occupation metrics. This index, which is based on the scale proposed by the Spanish Society of Epidemiology, categorizes family SES into five distinct groups: lower, upper lower, lower middle, upper middle, and upper [83].

Participants will complete a self-administered questionnaire to provide information on medication and family history of mental or cardiovascular disorders, as well as demographic characteristics, including birth date, sex at birth, gender, migrant status, marital status, type of housing, and, for female participants, menstrual status, pregnancy, and breastfeeding.

Anthropometric measures and body composition: Body weight and other body composition variables, such as body fat percentage, fat mass and lean mass, will be assessed using a Tanita MC-780 MA-N analyzer (Tokyo, Japan). Height will be measured to the nearest 0.1 cm using a wall-mounted stadiometer (Seca^®^ 213, Hamburg, Germany). Body composition and height will be measured in duplicate, and the average of the two measurements will be used for analysis. The body mass index (BMI) will be calculated using the Quetelet formula (weight [kg]/height [m^2^]). Waist circumference will be measured by the average of three determinations at the midpoint between the lowest rib and the superior border of the iliac crest following normal exhalation, with participants removing belts or clothing that could alter body shape without applying external pressure. Additionally, dual-energy X-ray absorptiometry (DXA) will be used to assess lean mass, fat mass, bone mineral content (kg), and bone mineral density (g/cm^2^) (Lunar iDXA, GE Medical Systems, Madison, WI, USA). All scans were processed using Physician’s Viewer, APEX System Software Version 3.1.2 (Worthington, MN, USA), and analyses were performed with enCoreTM 2008 software, version 12.30.008 (GE Healthcare, Madison, WI, USA). Instrument accuracy will be verified daily prior to each scanning session using the GE Lunar calibration phantom, following the manufacturer’s recommendations. All measurements will be obtained at high resolution by a trained researcher using a standardized protocol.

Food consumption and nutrient intake: Two methods will be used to assess dietary behavior. First, a 137-item semiquantitative food frequency questionnaire (FFQ) from the PREDIMED Plus study [84] will be administered to assess participants’ habitual dietary intake over the previous year. This FFQ has demonstrated good reproducibility (ICC: 0.63–0.90) and acceptable validity (ICC: 0.40–0.84) for food group assessment in an elderly Spanish Mediterranean population [84]. The validated tool captures the frequency and portion size of a wide variety of foods, including fruits, vegetables, legumes, cereals, dairy products, meats, fish, eggs, nuts, oils, and beverages such as wine, coffee, and soft drinks. The data collected will allow the estimation of energy intake, macronutrients (carbohydrates, proteins, fats), micronutrients (vitamins and minerals), fiber, and bioactive compounds such as polyphenols. Food composition data will be collected from the Spanish Food Composition Database [85] to derive nutrient compositions and estimate total energy intake.

Adherence to the Mediterranean diet: Adherence to the Mediterranean diet (MedDiet) and its specific components will be measured with the Mediterranean Diet Adherence Screener (MEDAS), a Spanish-validated questionnaire that consists of 14 questions [67]. The MEDAS has demonstrated acceptable concurrent validity against a validated FFQ (r = 0.52, ICC = 0.51) in Spanish adults at high cardiovascular risk [86]. The participants will be provided with the MEDAS to complete a tablet, including 12 questions about the frequency of food intake (i.e., olive oil, vegetables, fruit, red meat, animal fats, carbonated beverages, red wine, fish/seafood, nuts, commercial food, and traditional sauces) and 2 questions about food intake habits (i.e., preferred cooking fat used and type of meat consumed) considered characteristic of the Spanish MedDiet. Each question offers two response options, with corresponding scores of 0 (no adherence to the MedDiet component) or 1 (adherence to the MedDiet component).

Specifically, participants are awarded 1 point for adhering to specific dietary components, which includes the use of olive oil as the primary source of fat for cooking, which favors white meat over red meat and consumes the following on a daily or weekly basis: (i) 4 or more tablespoons (~13.5 g per tablespoon) of olive oil per day; (ii) 2 or more servings of vegetables per day; (iii) 3 or more servings of fruit per day; (iv) less than 1 serving of red meat or sausages per day; (v) less than 1 serving of animal fat per day; (vi) less than 1 cup (~100 milliliters) of sugar-sweetened beverages per day; (vii) 7 or more servings of red wine per week; (viii) 3 or more servings of pulses per week; (ix) 3 or more servings of fish per week; (x) fewer than 2 commercial pastries per week; (xi) 3 or more servings of nuts per week; and (xii) 2 or more servings per week of a dish featuring a traditional sauce made of tomatoes, garlic, onion, or leeks sautéed in olive oil.

The final score is the sum of each item (0–14), where a higher score indicates higher MedDiet adherence. The MEDAS score can be categorized as follows: scores < 9 indicate low adherence to the MedDiet, and scores from 9 to 14 indicate good adherence to the MedDiet [87].

Screen time: Screen time will be assessed using a self-administered questionnaire in which participants will report the number of hours they usually spend watching television, using a mobile phone, computer (for work), or other electronic devices (for leisure use) on both weekdays and weekends.

Social media addiction: Social media addiction will be measured using the short-form Social Media Addiction Scale (ARS-6), a six-item self-report instrument designed to capture core components of addictive behavior, including salience, mood modification, tolerance, conflict, and relapse, adapted to the social media context. The participants respond on a five-point Likert scale ranging from “never” to “always”, with higher scores indicating a greater risk of social media addiction [88]. This scale has been validated in adolescent and university populations, demonstrating excellent psychometric properties, including high internal consistency (Cronbach’s α = 0.923–0.950; McDonald’s ω = 0.916–0.960) [88,89].

Problematic substance use: The Alcohol, Smoking and Substance Involvement Screening Test (ASSIST) [90] will be administered to evaluate participants’ risk of substance-use disorders and associated health, social, and legal consequences. This instrument, developed by the World Health Organization (WHO), enables the assessment of involvement across multiple substance categories (including alcohol, tobacco, and illicit drugs) by means of structured items addressing lifetime use, frequency of use in the past three months, craving intensity, failed obligations, and concerns expressed by others. Risk classifications follow the original ASSIST thresholds: for alcohol, 0–10 points indicate low risk, 11–26 points indicate moderate risk, and 27 points or more indicate high risk; for tobacco and all other substances, 0–3 points indicate low risk, 4–26 points indicate moderate risk, and 27 points or more indicate high risk. The Spanish version of the ASSIST has demonstrated adequate psychometric properties, with high internal consistency (Cronbach’s α = 0.86–0.97 across substances) [91].

Physical activity: The short form of the International Physical Activity Questionnaire (IPAQ) [92] will be used to quantify participants’ physical activity levels. This seven-item instrument provides a comprehensive assessment of vigorous-intensity activity, moderate-intensity activity, walking, and sedentary time during the preceding week. Following the official scoring protocol, reported activity durations for each activity category will be converted into metabolic equivalent of task minutes per week (MET-min/week), enabling classification of individuals into low, moderate, or high physical activity levels. Low activity level includes individuals who do not meet moderate or high criteria (inactive); moderate level includes those engaging in ≥3 days of vigorous activity (20 min/day), ≥5 days of moderate-intensity activity or walking (30 min/day), or any combination totaling ≥600 MET-min/week; high level includes those with ≥3 days of vigorous activity totaling ≥1500 MET-min/week, or ≥7 days of any combination of walking, moderate, or vigorous activity totaling ≥3000 MET-min/week. The IPAQ has demonstrated a good reliability coefficient for total physical activity (r = 0.82, *p* < 0.05), vigorous activity (r = 0.79, *p* < 0.05), moderate activity (r = 0.83, *p* < 0.05), and time spent walking (r = 0.73, *p* < 0.05) within Spanish-speaking adult populations, including samples of university students [92,93].

Muscular strength: Upper-body muscle strength (handgrip strength in kilograms) will be assessed following a 2 min warm-up consisting of joint mobilization and static stretching. The maximal isometric handgrip strength will be measured using a calibrated dynamometer (TKK 5401 Grip-D, Takeya, Tokyo, Japan) [94] with the grip adjusted to the participant’s dominant hand size, ensuring that the second joint of the index finger forms approximately a right angle. Hand size, defined as the distance from the tip of the thumb to the fifth digit at maximum extension, will be measured prior to testing.

The participants stand upright with their feet’ shoulder width apart, with their arms extended and slightly away from the body and perform two maximal squeezes per hand for 3–5 s, with 10 s rest intervals between attempts. Prior to testing, the procedure and grip technique will be demonstrated, and participants will perform practice squeezes to ensure proper technique. Excessive movements are prohibited, and maximal effort is encouraged throughout. Handgrip strength will be recorded in kilograms, with the highest value used for analysis.

The standing long jump test will be used to assess lower-body muscular strength [95]. The participants stand behind a designated line with their feet approximately shoulder width apart and jump forward with both feet simultaneously, aiming to achieve maximum distance. The distance will be measured in centimeters from the starting line to the posterior edge of the heels upon landing. Each participant will perform three attempts, and the longest jump will be recorded for subsequent analysis.

Self-reported fitness: Additionally, physical fitness will be assessed using the International Fitness Scale (IFIS) [96,97], a validated self-report questionnaire. The participants will be asked to rate their perceived overall fitness, cardiorespiratory fitness, muscular strength, speed/agility, and flexibility on a five-point Likert scale from “very poor” to “very good”. The IFIS has demonstrated reliability and validity in Spanish-speaking university populations, providing a feasible tool to evaluate subjective fitness levels with a Cronbach’s alpha of 0.90 [98] and an intraclass correlation coefficient of 0.947 [99].

Blood pressure: Blood pressure will be measured following standardized procedures. The participants will be instructed to empty their bladder prior to measurement and will then rest in a seated position for 5 min before assessment. Two BP measurements will be taken on the same arm with a 3 min interval between readings. A third measurement will be obtained if (1) the systolic blood pressure (SBP) in any measurement exceeds 130 mmHg or if (2) the difference between the two measurements is >10 mmHg for either SBP or diastolic blood pressure (DBP). The average of the two valid measurements will be used for analysis. Measurements will be obtained using a validated automated device (OMRON HEM-907, Omron Healthcare UK Ltd., Milton Keynes, UK), and the mean of the two readings will be used for analysis.

Blood biochemical parameters: Fasting blood samples will be collected from the cubital vein between 08:15 and 09:00 h following at least 12 h of overnight fasting. These samples will be used to determine basic hemograms; lipid profiles, including total cholesterol, high-density lipoprotein cholesterol (HDL-c), low-density lipoprotein cholesterol (LDL-c), and triglycerides; glycemic metabolism markers, including fasting serum glucose, insulin, and glycated hemoglobin (HbA1c); selected vitamins (vitamin D, vitamin E, vitamin B12, and folic acid); essential minerals (iron and magnesium); and inflammatory markers, including high-sensitivity C-reactive protein (hs-CRP) and interleukin 6 (IL-6). All analyses will be performed using standardized laboratory methods under strict quality control procedures to ensure accuracy and reliability.

Brain-derived neurotrophic factor (BDNF): BDNF has received increasing attention because of its potential correlation with mental health [28]. Blood levels of BDNF will be measured to assess neurotrophic support in participants. Venous blood samples will be collected in tubes to separate the serum with gel following an overnight fast to minimize circadian and dietary variability. The samples will be immediately centrifuged at 3000 g for 10 min at ambient temperature, and the serum will be aliquoted and stored at −80 °C until analysis. BDNF concentrations will be quantified using a commercially available enzyme-linked immunosorbent assay (ELISA) kit (Aviscera Bioscience, Santa Clara, CA, USA, INC SK00752-01C) following the manufacturer’s protocol. All assays will be performed in duplicate, with intra-and inter-assay coefficients of variation maintained below 10%. The results will be expressed in pg/mL and analyzed as continuous variables in subsequent statistical analyses.

### 2.11. Data Management and Statistical Analysis Plan

The principal investigator will have exclusive responsibility for the data and will retain an offline copy on a secure hard drive. The anonymized data will be stored in an open-access repository (Zenodo.org) and may be shared with other researchers upon reasonable request.

The initial analytical approach will comprise descriptive statistics and data visualization to perform an exploratory assessment of the dataset, including an evaluation of bivariate associations between the predictor variables (nut consumption doses) and the outcome variables (mental health parameters). This stage will assess potential baseline differences among the three groups (CG, IG-30, and IG-60).

Subsequently, assumptions of normality and homoscedasticity for the outcome variables will be examined to ascertain the suitability of employing a repeated-measures mixed-effects model to characterize the intervention relationship between nut consumption and mental health indicators. Interpretation will focus on the magnitude and statistical significance of the fixed effects coefficients, thereby determining whether nut consumption is associated with changes in mental health outcomes and whether these associations vary across study intervention groups.

The role of BDNF will also be analyzed, examining associations between BDNF changes and mental health measures through correlation analysis at each timepoint, mediation analyses using structural equation modeling to test whether BDNF mediates the relationship between nut consumption and mental health, including cognitive improvements, and dose–response analyses across study arms. Secondary exploratory analyses will include responder analyses (BDNF increase), time-course analyses using linear mixed models, and interaction analyses examining moderators such as baseline BDNF levels and mental health status. All BDNF-cognition analyses will be designated as secondary/exploratory with appropriate adjustments for multiple comparisons and covariates, including age, sex, baseline cognitive performance, physical activity, and dietary quality.

The intention-to-treat (ITT) analysis will serve as the primary analysis to preserve randomization benefits and provide a conservative estimate of treatment effects, whereas the per-protocol (PP) analysis will serve as a sensitivity analysis to assess efficacy under optimal adherence conditions. The ITT analysis will include all randomized participants in their assigned groups regardless of adherence, with missing data handled using multiple imputation or mixed-effects models that accommodate incomplete observations. The PP analysis will include only participants who completed the intervention according to protocol specifications, with adherence thresholds defined a priori (e.g., self-reported consumption of at least 80% of nut doses assigned, and 5–15% increased blood concentrations of α-tocopherol) [61].

The interpretation of clinical significance will be based on established minimal clinically important differences (MCID) for each outcome measure. For example, for the BDI-II, we will apply a 17.5% reduction as determined by Button et al. (2015) [100], who found that this represents the threshold at which patients report feeling “better” based on anchor-based methods. We will also evaluate whether participants transition between established severity categories (minimal: 0–13; mild: 14–19; moderate: 20–28; severe: 29–63) [63]. For the GAD-7, we will use the 4-point MCID threshold established by Toussaint et al. (2020) [101] to assess whether participants cross the clinical cut-off of ≥10 points, indicating clinically significant anxiety [66]. Given the lack of consensus on the MCID for the PSS-10, we will interpret effect sizes of Cohen’s d ≥ 0.5 as clinically relevant [57] and contextualize changes using reference values for the target population [102]. We recognize that in preventive nutritional interventions targeting non-clinical populations, small but consistent effects may have beneficial public health impacts [103], particularly given that dietary modifications represent accessible and low-risk interventions.

The results will be considered statistically significant at the α = 0.05 level. In the presence of statistically significant effects, post hoc analyses will be conducted using a pairwise comparison test to delineate specific contrasts between each intervention group and the control arm. Furthermore, statistical and clinical significance will be distinguished in our discussion, considering both the magnitude of effects and their potential implications for guidelines recommending nut consumption as a strategy to prevent or improve mental health outcomes. Therefore, when the estimators of associations do not reach statistical significance due to the small difference between the intervention and control groups, the results will be presented for their descriptive value and to inform future RCTs exploring the relationship between nuts and mental health. Although *p*-values are relevant, changes that are not statistically significant can still be clinically meaningful [104].

### 2.12. Limitations and Contingency Plan

#### 2.12.1. Limitations

The study uses a single-blind design because participants cannot be blinded to nut consumption. This may introduce expectancy bias, but it is mitigated by blinding outcome researchers. Primary mental health outcomes rely on self-report questionnaires and are therefore subject to reporting biases. However, validated instruments and complementary objective measures, such as cognitive tests and biomarkers, are included. Adherence cannot be directly observed in free-living conditions. Monitoring through biweekly contact and determination of vitamin E blood concentrations at each visit improves, but does not guarantee, compliance and does not prevent contamination of the control group. The sample consists of university students aged 18–24 years from a single campus. This limits generalizability but enhances internal validity in an understudied population. The study may be underpowered for small effects, secondary outcomes, or subgroup analyses, particularly if dropout exceeds the anticipated 20%. Finally, the 6-month intervention and follow-up period may be insufficient to assess long-term or delayed effects.

The extensive assessment protocol may increase participant burden and the risk of attrition. Although a validated FFQ will be used, dietary assessment remains imprecise and is subject to underreporting and recall bias. Additionally, some control group participants may live with participants from other study groups, which makes complete prevention of contamination unrealistic. While the CG will be instructed to avoid nuts from any source and the IG-30 and IG-60 groups will be instructed to avoid nuts from non-study sources, accidental or intentional nut consumption may still occur.

Finally, academic stressors (e.g., exams, tight assignment deadlines) and seasonal variations may affect mental health and thereby influence the study associations.

#### 2.12.2. Contingency Plan

- Recruitment and retention: If enrollment targets are not met, recruitment efforts will expand across the university. These efforts will use multiple communication channels, classroom presentations, and student organizations. If necessary, eligibility criteria may be slightly adjusted with ethical approval. If the number of students who drop out exceeds expectations, retention strategies will be intensified. These strategies include increased contact, flexible scheduling, reminders, small incentives, and feedback on selected results. Participants who are unable to continue in person will be encouraged to complete all self-reported assessments remotely to support intention-to-treat analyses.
- Intervention delivery: A backup nut supply and alternative suppliers will be maintained. Any adverse reactions will lead to immediate discontinuation and medical referral. Participants for whom poor adherence is detected during the intervention will receive individualized support and encouragement to remain in the study, regardless of their level of adherence.
- Data collection and analysis: Backup equipment and rescheduling windows will address equipment failure, technician strikes, and other unpredictable circumstances. Missing data, except for covariates, will be handled using multiple imputation and mixed-effects models. Sensitivity analyses will be performed to assess potential bias.
- External and ethical considerations: In the event of public health emergencies, such as the COVID-19 pandemic that emerged in 2020, assessments will shift to remote formats where feasible. Seasonal and academic calendar effects will be addressed analytically. Any protocol amendments will receive prior ethical approval, and robust procedures are in place to manage data security incidents.
- Monitoring and quality assurance: Study progress, adherence, retention, and data quality will be monitored monthly by the study coordinator and reviewed quarterly by the principal investigator. Serious adverse events or unexpected issues will prompt immediate review and, if needed, consultation with the ethics committee. Vitamin E (α-tocopherol) levels will be measured in all participants at baseline, 3 months, and 6 months, along with dietary intake assessments. Unauthorized nut consumption in the control group will be detected through either increases in vitamin E levels > 5% from baseline or self-reported nut intake in dietary recalls.
- Academic stressors and seasonal variations: The following strategies will be adopted to address confounding by academic-related stressors (e.g., examination periods, high academic demands) and seasonal factors: (1) randomization, to balance exposure to stressful periods across groups; (2) tracking assessment dates, to categorize examination periods and seasons; (3) repeated-measures mixed-effects models, adjusting for these factors as covariates; and (4) sensitivity analyses, to test the robustness of findings. Furthermore, stress is directly monitored as a primary outcome using the PSS-10 scale and may reflect partially academic-related stress symptoms.

### 2.13. Dissemination and Communication Plan

The findings will be disseminated through high-impact open-access peer-reviewed publications and presentations at scientific events. In addition, they will be shared with the study population and the university community through talks and posters, and communicated to the general public via media channels such as radio, social media, and other outreach platforms.

## 3. Discussion

The Nuts4Brain-RCT has been designed to provide experimental evidence on the effects of specific doses of regular nut consumption on mental health-related outcomes in young adults. While the cardiovascular benefits of nuts are well established, with 30 g/day generally considered the minimum effective dose, the potential dose–response relationship for mental health outcomes remains unexplored. Furthermore, this study addresses a critical gap in the literature, which is largely limited to observational studies or trials targeting cognitive decline in older populations. Young adulthood represents a developmental window marked by pronounced psychosocial transitions and peak onset of mental disorders [42,105]. By focusing on this specific age group, the Nuts4Brain-RCT aims to support the development of scalable nutritional interventions.

We hypothesize that nut consumption will modulate key biological pathways, particularly by increasing fiber consumption (which improves the gut microbiota status and gut–brain axis signaling); providing essential minerals such as magnesium and zinc (cofactors in neurotransmitter synthesis); supplying unsaturated fatty acids (which maintain neuronal membrane integrity and reduce inflammatory responses); delivering antioxidants, including vitamin E, polyphenols, and selenium (which combat cellular oxidative damage); and providing L-arginine (a nitric oxide precursor that supports cerebrovascular function), thereby reducing oxidative stress and systemic inflammation, both of which are implicated in the pathophysiology of mental health disorders.

Specifically, daily consumption of 30 g of mixed nuts for six months is expected to result in a decrease in the frequency, severity, and number of depressive symptoms and a reduction in anxiety and stress levels, alongside improvements in mental well-being, cognitive function, and sleep quality. The participants who will consume 60 g/day are anticipated to demonstrate a modest increase in these benefits, reflecting a potential dose–response effect. This hypothesis is based on biological plausibility, suggesting that a higher intake of certain compounds (e.g., polyphenols and omega-3 fatty acids) may exert stronger anti-inflammatory, antioxidant and neuroprotective effects required to modulate brain function [19,23]. Additionally, the intervention is expected to improve overall diet quality, maintain body weight (with possible minor reductions in body fat percentage and waist circumference), and enhance metabolic health, including improved lipid profiles, lower blood glucose levels, and reduced inflammatory markers, compared with those of the control group.

This protocol presents several key strengths. First, its randomized controlled design with a dose–response approach addresses a critical evidence gap regarding the “optimal dose” of nut consumption for enhancing mental well-being. Second, the study adopts a multidisciplinary assessment strategy that integrates validated psychometric scales (BDI-II, GAD-7), comprehensive neuropsychological evaluations, and biochemical validation (BDNF). Third, the intervention extends over a six-month period, providing a sufficiently long timeframe to observe meaningful effects. In addition, a fourth visit will be carried out six months after the end of the intervention to evaluate whether nut consumption and its potential effects persist over time. Fourth, adherence will be rigorously monitored through the provision of prepacked nut portions and objective verification via plasma vitamin E levels, thereby increasing compliance in a manner that overcomes challenges typically associated with dietary interventions. Finally, relevant variables (habitual dietary intake, physical activity, fitness, etc.) will be examined for moderating, mediating, or confounding effects.

Limitations include the inability to blind participants due to the whole-food nature of the intervention, which may introduce performance or expectation bias. To minimize these biases, a multi-layer strategy will be implemented. First, allocation will be strictly concealed using opaque, sealed envelopes until baseline assessments are completed. Following randomization, outcome assessors and data analysts will remain blinded to group allocation throughout the trial. During the intervention period, compliance will be monitored via blood vitamin E concentrations. At assessment points, self-reported mental health measures (BDI-II, GAD-7) will be complemented with objective cognitive assessments (NIH Toolbox) and biological markers (BDNF, inflammatory markers, cortisol). Together, these measures help mitigate expectancy effects and strengthen the trial’s internal validity. The recruitment of university students enhances sample homogeneity but may also restrict the generalizability of the findings to broader young adult populations. Finally, although the study is powered to detect medium effect sizes, mental health outcomes are multifactorial and may be influenced by unmeasured genetic or environmental factors.

## 4. Conclusions

The Nuts4Brain-RCT will generate high-quality evidence on whether nut consumption can be considered a nonpharmacological strategy for promoting mental health in young adults. If effective, the findings could inform public health strategies and evidence-based dietary recommendations aimed at reducing the burden of mental health disorders in this population.

## Figures and Tables

**Table 1:** Data collection methods and measurement protocols for the study variables in the Nuts4Brain-RCT study.

Outcomes			
Group of variables	Outcomes	Scales or measurement procedures	Collection methods
Primary mental health outcome measures	Depression	BDI-II	Tablet self-report
	Anxiety	GAD-7 questionnaire	
	Stress	PSS-10	
	Cognitive functioning	Flanker, DCCS, PSM, LSWM, PC	Tablet application guided by researcher
Secondary mental health outcome variables	Risk of eating disorders	SCOFF questionnaire	Tablet self-report
	Body image perception	SFS	
	Resilience	CD-RISC-10	
	Quality of Life	SF-12 Health Survey	
	Sleep quality	PSQI	
Potential confounding, mediating, and moderating factors			
Socioeconomic level	Socioeconomic Status	SES	Tablet self-report
Demographic features	Birth date, sex, gender, marital status, type of housing, and, for female participants, breastfeeding, and menstrual status.	Ad hoc questionnaire	
Medical history	Medication and family history of mental or cardiovascular disorders		
Anthropometry	Height, weight, waist circumference	Anthropometry tests	Physical measurement
Body composition	Fat mass, lean mass	Bioelectrical impedance analysis	
	Fat mass, lean mass, bone mineral density, bone mineral content	Dual-energy X-ray absorptiometry	
Lifestyle behaviors	Food consumption and nutrient intake	Food frequency questionnaire-137	Paper self-report
	Nut consumption	Ad hoc questionnaire	Tablet self-report
	Adherence to the Mediterranean diet and its specific components	MEDAS questionnaire	
	Screen time	Ad hoc questionnaire	
	Social media addiction	ARS-6	
	Problematic substance use	ASSIST	
	Physical activity	IPAQ	
Health-related variables	Cardiorespiratory fitness, agility and speed, muscular strength, flexibility, overall physical fitness	IFIS	
	Muscular strength	Standing long jump and handgrip strength tests	Physical measurement
	Blood pressure	Blood pressure test	
	BDNF	Blood tests ELISA	Blood biochemical parameters
	Hemogram, total cholesterol, HDL-c, LDL-c, triglycerides, glucose, insulin, HbA1c, vitamin D, vitamin E, vitamin B12, folic acid, iron, magnesium, IL-6 and hs-CRP.	Blood tests	

Abbreviations: ARS-6, Social Media Addiction Scale–6 Items; ASSIST, Alcohol, Smoking and Substance Involvement Screening Test; BDI-II, Beck Depression Inventory-II; BDNF, Brain-Derived Neurotrophic Factor; CD-RISC-10, Connor–Davidson Resilience Scale–10 Items; DASS-21, Depression, Anxiety and Stress Scale–21 Items; DCCS, Dimensional Change Card Sort Test; Flanker, Flanker Inhibitory Control and Attention Test; GAD-7, Generalized Anxiety Disorder–7 Items; HbA1c, glycated hemoglobin; HDL-c, high-density lipoprotein cholesterol; hs-CRP, high-sensitivity C-reactive protein; IFIS, International Fitness Scale; IL-6: interleukin 6; IPAQ, International Physical Activity Questionnaire; LDL-c, low-density lipoprotein cholesterol; LSWM, List Sorting Working Memory Test; MEDAS, Mediterranean Diet Adherence Screener; PC, Pattern Comparison Processing Speed Test; PSM, Picture Sequence Memory Test; PSQI, Pittsburgh Sleep Quality Index; SCOFF, Sick, Control, One, Fat, Food; SES, Family Socioeconomic Status; SF-12, 12-Item Short Form Health Survey; SFS, Standard Figural Stimuli.

## Data Availability

No new data were created or analyzed in this study.

## Abbreviations

The following abbreviations are used in this manuscript:

AMPM	Automated Multiple-Pass Method
ARS-6	Adolescent Resilience Scale–6 Items
ASSIST	Alcohol, Smoking and Substance Involvement Screening Test
BDI-II	Beck Depression Inventory-II
BDNF	Brain-Derived Neurotrophic Factor
BMI	Body Mass Index
CD-RISC-10	Connor–Davidson Resilience Scale–10 Items
CG	Control Group
COVID-19	Coronavirus Disease 2019
DBP	Diastolic Blood Pressure
DCCS	Dimensional Change Card Sort Test
DXA	Dual-Energy X-ray Absorptiometry
ELISA	Enzyme-Linked Immunosorbent Assay
FFQ	Food Frequency Questionnaire
GAD-7	Generalized Anxiety Disorder–7 Items
HbA1c	Glycated Hemoglobin
HDL-c	High-Density Lipoprotein Cholesterol
HRQoL	Health-Related Quality of Life
hs-CRP	High-Sensitivity C-Reactive Protein
IFIS	International Fitness Scale
IG-30	Intervention Group 30 g/day
IG-60	Intervention Group 60 g/day
IL-6	Interleukin 6
IPAQ	International Physical Activity Questionnaire
ITT	Intention-to-Treat
LDL-c	Low-Density Lipoprotein Cholesterol
LSWM	List Sorting Working Memory Test
MCS	Mental Component Summary
MEDAS	Mediterranean Diet Adherence Screener
MedDiet	Mediterranean Diet
MET	Metabolic Equivalent
MUFAs	Monounsaturated Fatty Acids
NIH	National Institutes of Health
PC	Pattern Comparison Processing Speed Test
PCPST	Pattern Comparison Processing Speed Test
PCS	Physical Component Summary
PP	Per-Protocol
PSM	Picture Sequence Memory Test
PSMT	Picture Sequence Memory Test
PSS-10	Perceived Stress Scale–10 Items
PSQI	Pittsburgh Sleep Quality Index
PUFAs	Polyunsaturated Fatty Acids
RCT	Randomized Controlled Trial
SBP	Systolic Blood Pressure
SCOFF	Sick, Control, One, Fat, Food
SES	Socioeconomic Status
SF-12	12-Item Short Form Health Survey
SFS	Standard Figural Stimuli
SPIRIT	Standard Protocol Items: Recommendations for Interventional Trials
UCLM	University of Castilla-La Mancha
WHO	World Health Organization
